# Prevalence of Malocclusion and Orthodontic Treatment Needs in the Mixed Dentition Period of the Kashmiri Population Using the Index of Orthodontic Treatment Needs: A Hospital-Based Study

**DOI:** 10.7759/cureus.115161

**Published:** 2026-08-25

**Authors:** Nadia Irshad, Nazia Lone, Insha Showkat, Sana Farooq, Hilal Hela, Navneet Kour, Mohsin Sidiq

**Affiliations:** 1 Pediatric Dentistry, Government Dental College and Hospital, Srinagar, IND; 2 Pediatric Dentistry, Government Dental College and Hospital Srinagar, Srinagar, IND; 3 Prosthodontics Crown and Bridge, Government Dental College and Hospital, Srinagar, IND; 4 Periodontics, Sher-i-Kashmir Institute of Medical Sciences, Srinagar, IND

**Keywords:** index of orthodontic treatment needs, kashmiri population, malocclusion, mixed dentition, : prevalence

## Abstract

Background

Malocclusion has a huge impact on individuals as well as society in terms of discomfort, quality of life, and social and functional limitations. Estimating the need for orthodontic treatment in the population is crucial for planning orthodontic management and monitoring oral health programs. Therefore, the present study aimed to assess the prevalence of malocclusion and orthodontic treatment needs among children of the Kashmiri population.

Material and methodology

A total of 9,268 children aged between six and 12 years visiting the outpatient department (OPD) of the department were assessed to determine the prevalence of malocclusion using the Index of Orthodontic Treatment Need (IOTN).

Statistical analysis

The collected data were entered into a spreadsheet and analyzed using appropriate statistical software. Descriptive statistics were used to calculate frequencies and percentages, and the Chi-square test was used to assess the association between malocclusion traits and orthodontic treatment need. The statistically significant association between malocclusion traits and orthodontic treatment need was set at p < 0.001.

Results

Out of the study population, 1,848 children had different traits of malocclusion; the overall prevalence of malocclusion was 19.9%, and the most common malocclusion traits were increased overjet (33.5%) and crowding (29.2%).

Conclusion

The authors concluded that malocclusion is not a single entity but rather a collection of situations, each in itself constituting a problem, and can be complicated by a multitude of genetic and environmental causes. The need to implement preventive and interceptive orthodontic care, such as habit-breaking appliances, caries prevention, etc., is of utmost importance.

## Introduction

Malocclusion refers to misalignment or incorrect relationship between the teeth of the two dental arches when they approach each other as the jaws close. Malocclusion is considered to be the most common oral health problem worldwide, along with dental caries, dental fluorosis, and gingivitis [1].

It is a multifactorial oral health issue caused by general factors such as congenital defects, heredity, nutritional deficiencies, and abnormal pressure habits, particularly thumb sucking. Malocclusion can also occur due to factors located in the dental arch such as anomalies of tooth size, shape, supernumerary teeth, dental caries, and premature loss of primary teeth [2].

Various indices have been used from time to time in order to categorize medical and dental disorders/diseases for the purpose of epidemiology, research, and to distribute patients into categories of treatment need. An index can be defined as a numerical value describing the relative status of a population on a graduated scale with defined upper and lower limits, which is designed to permit and facilitate the comparison with other populations classified under the same method and criteria. In the orthodontic context, an index is used to describe a rating or categorizing system that assigns an alphanumeric label or numeric score to a person’s occlusion. Different orthodontic indices have been proposed to provide information on the prevalence of malocclusions and objectively quantify the severity of the various features of malocclusion [3].

Malocclusion has a huge burden and impact on individuals and society, and in recent years several efforts have been put forward on measuring the prevalence and severity of malocclusion and orthodontic treatment need worldwide. Assessing the resources required to meet the orthodontic treatment demand and rationally plan for their provision requires population-level data [4].

Many developing countries, including India, are struggling to eradicate medical and dental problems. The main reason behind its limited success is an inadequate implementation of different preventive oral healthcare programs and strategies, which in turn needs a sound base of epidemiological data. Epidemiological studies related to occlusion and malocclusion not only help in orthodontic treatment planning and evaluation of dental services but also offer a valid and particular research tool for ascertaining the role of distinct environmental and genetic factors in the causation of malocclusion. Indices of orthodontic treatment are used in screenings and epidemiological studies to identify the priority of treatment [5]. There is a deficiency regarding malocclusion and orthodontic treatment needs during the mixed dentition period of children in Kashmir; the mixed dentition stage is a critical period for early diagnosis and interceptive orthodontic intervention. Furthermore, it is emphasized that Government Dental College Srinagar is the only tertiary care dental setup in Kashmir, and it can provide the baseline regional data for clinical practice and future epidemiological research. Therefore, the present hospital-based cross-sectional study was undertaken to evaluate the prevalence of malocclusion trait and determine orthodontic treatment needs using the dental health component (DHC) of the Index of Orthodontic Treatment Need (IOTN) among children aged six to 12 years attending the outpatient department of Government Dental College and Hospital, Srinagar, Kashmir.

## Materials and methods

Study design and setting

A hospital-based cross-sectional study was conducted in the outpatient Department of Paediatric and Preventive Dentistry, Government Dental College (GDC) and Hospital, Srinagar (Kashmir). The study was carried out over a defined study period (for a period of one year) after obtaining approval from the Institutional Ethics Committee (EC/NEW/INST/2023/4133). Written informed consent was obtained from the parents of the study population.

Study population

The study included children aged six to 12 years presenting to the outpatient department during the study period. This age group was selected to represent the mixed dentition period, which was critical for early identification of developing malocclusions.

Sample size

As we know, a priori sample size calculation is generally recommended for observational studies. However, the present study was designed as a hospital-based cross-sectional study using consecutive sampling, in which all eligible children aged six to 12 years attending the outpatient department during the one-year study period were included. The final sample size (n = 9,268) was therefore determined by the total number of eligible participants presenting during the one-year study period rather than by a predetermined sample size calculation. Given the very large sample obtained, we believe that the study had sufficient precision to estimate the prevalence of malocclusion and orthodontic treatment needs. Moreover, GDC Srinagar is the only tertiary care hospital in the entire Kashmir Valley, and the outpatient department (OPD) is much more likely to have a significant sample size.

Inclusion and exclusion criteria

Children aged between six and 12 years who were in the mixed dentition period and attended the outpatient department were considered eligible for inclusion in the study. Only those children who were accompanied by their parents or guardians and for whom informed consent was obtained were enrolled. Children with a history of previous or ongoing orthodontic treatment were excluded from the study. Additionally, children presenting with craniofacial anomalies such as cleft lip and palate, those suffering from systemic conditions or syndromes known to affect craniofacial growth, children who were uncooperative or could not be adequately examined, and children with self-correcting anomalies or trauma to anterior teeth were also excluded. Also, children having anterior open-bite due to early exfoliation of anterior deciduous teeth, partially erupted permanent teeth, or grossly decayed anterior teeth were also excluded and not counted as a malocclusion trait.

Data collection and clinical examination

Clinical examinations were carried out by two trained and calibrated pedodontists using sterile mouth mirrors and probes under adequate illumination. The occlusal characteristics were recorded with the child (patient) seated in a dental chair in centric occlusion.

Assessment of malocclusion and orthodontic treatment need

Malocclusion and orthodontic treatment need were assessed using the IOTN [6]. The index consists of two components: a) dental health component (DHC) and aesthetic component (AC).

Dental Health Component (DHC)

This component evaluates the severity of malocclusion based on specific occlusal traits such as crowding, open bite, overjet, crossbite, and impacted teeth. It can be assessed clinically without relying on subjective judgments of appearance. This component identifies malocclusions and their treatment and is easier to standardize across large populations and examiners.

Aesthetic Component (AC)

Assesses the aesthetic impairment caused by malocclusion using a standardized 10-point photographic scale. This component evaluates the aesthetics, including the psychosocial impact of malocclusion in addition to treatment need.

As it was an OPD-based study and the primary objective was to determine the orthodontic treatment need, only DHC was included in the study, as it is more reproducible and standardizes assessment suitable for epidemiological studies and minimizes the influence of subjective aesthetic perception associated with the aesthetic component. Based on the IOTN scores, children were categorized into different grades reflecting little/no need, borderline need, or definite need for orthodontic treatment.

Inter-examiner calibration and reliability

Data collection was done by two examiners, and before data collection, both examiners underwent calibration exercises or sessions to ensure consistency in scoring of data. To assess examiner reliability, 20 participants were randomly selected, and both examiners individually calculated the score. The inter-examiner reliability (Cohen's kappa values) at that time (baseline) was found to be (κ = 0.931). The same selected participants were re-examined after an interval of two to three weeks to assess intra-examiner reliability. The intra-examiner reliability for examiner one was (κ =0.863), and for examiner two was (κ =0.860). The inter-examiner reliability after two to three weeks was (κ =0.930), demonstrating almost perfect agreement and high reliability of the IOTN scoring. This inter-examiner reliability was done to increase reproducibility, credibility, and enhance the quality of data.

Statistical analysis

The collected data were entered into a spreadsheet and analyzed using appropriate statistical software, SPSS version 21.0 (IBM Corp., Armonk, USA). Descriptive statistics were used to calculate frequencies and percentages of various malocclusion traits and orthodontic treatment needs. The Chi-square test was used to evaluate the association between malocclusion traits and orthodontic treatment needs. A p-value of < 0.05 was considered statistically significant. Results were presented in tables and charts, where applicable.

## Results

A total of 9,268 children aged six to 12 years in the mixed dentition period were examined. All participants fulfilled the inclusion criteria and were evaluated using the IOTN [6]. The age and sex distribution of the study population is presented in Table 1. Females constituted 58.7% (n = 1,084) of the children with malocclusion, while males accounted for 41.3% (n = 764). The mean age of the participants with malocclusion was 9.23 ± 1.52 years. Of the total study population, 1,848 children (19.9%) presented with one or more malocclusion traits, while 7,420 children (80.1%) exhibited normal occlusion (Table 2). Based on the dental health component of the IOTN, the majority of children demonstrated little or no need for orthodontic treatment. However, a considerable proportion required borderline or definite orthodontic intervention (Table 3). Among the 1,848 children identified with malocclusion, the majority fell under the definite treatment need category (Table 4). Increased overjet and crowding were the most frequently observed occlusal traits, followed by anterior crossbite and deep bite (Table 5).

**Table 1:** Distribution of study population by age group. Data were presented as numbers (N) and percentages (%) for categorical values, and Mean±Standard deviation (SD) with Standard Error (SE) for age.

		Frequency	Percentage	Mean SD
AGE	6 Years	100	5.41%	M = 9.23, SD = 1.52, SE = 0.035
	7 Years	48	2.59%	
	8 Years	476	25.75%	
	9 Years	492	26.62%	
	10 Years	352	19.04%	
	11 Years	196	10.60%	
	12 Years	184	9.95%	
SEX	Males	764	41.34%	M = 1.58, SD = 0.49, SE = 0.011
	Females	1084	58.65%	

**Table 2:** Prevalence of malocclusion among study population. Data were presented as numbers (N) and percentages (%).

Status	Number of Children	Percentage (%)
Normal occlusion	7,420	80.1
Presence of malocclusion	1,848	19.9
Total	9,268	100

**Table 3:** Distribution of orthodontic treatment need according to iotn (dental health component). Data were presented as numbers (N) and percentages (%). IOTN: Index of Orthodontic Treatment Need.

IOTN Category	Treatment Need	Number of Children	Percentage (%)
Grades 1–2	Little / No need	6,980	75.3
Grade 3	Borderline need	1,150	12.4
Grades 4–5	Definite need	1,138	12.3
Total		9,268	100

**Table 4:** Distribution of orthodontic treatment need among children with malocclusion (n = 1,848). Data were presented as numbers (N) and percentages (%). IOTN: Index of Orthodontic Treatment Need.

IOTN Category	Number of Children	Percentage (%)
Borderline need	710	38.4
Definite need	1,138	61.6
Total	1,848	100

**Table 5:** Distribution of common malocclusion traits Data was presented as number (N) and Percentage (%)

Malocclusion Trait	Number of Children	Percentage (%)
Increased overjet	620	33.5
Crowding	540	29.2
Anterior crossbite	310	16.8
Deep bite	248	13.4
Open bite	130	7.1
Total	1,848	100

Distribution of malocclusion traits and their association with orthodontic treatment need

Among the 1,848 children diagnosed with malocclusion, increased overjet was the most prevalent trait, affecting 620 children (33.5%), followed by crowding in 540 children (29.2%). Anterior crossbite was observed in 310 children (16.8%), while deep bite and open bite were present in 248 (13.4%) and 130 (7.1%) children, respectively (Table 6).

**Table 6:** Distribution of malocclusion traits and association with orthodontic treatment need (n = 1,848) Data was presented as N (%), associations were analysed using Chi Square (χ²) test and statistical significant p value was set at (p < 0.05)

Malocclusion Trait	Number (%)	Definite Treatment Need n (%)	χ² value	p-value
Increased overjet	620 (33.5)	470 (75.8)	198.4	<0.001
Crowding	540 (29.2)	350 (64.8)	122.6	<0.001
Anterior crossbite	310 (16.8)	180 (58.1)	64.9	<0.001
Deep bite	248 (13.4)	98 (39.5)	12.1	0.002
Open bite	130 (7.1)	90 (69.2)	28.7	<0.001

A statistically significant association was observed between the type of malocclusion trait and the need for orthodontic treatment as assessed by the IOTN dental health component (χ² = 421.6, p < 0.001).

Increased overjet (> 6 mm) showed a strong association with definite orthodontic treatment need (Grades 4-5), with a majority of affected children requiring comprehensive orthodontic intervention (p < 0.001). Crowding was significantly associated with both borderline and definite treatment need, depending on severity and space discrepancy (p < 0.001). Anterior crossbite demonstrated a significant association with borderline to definite treatment need, highlighting the importance of early interceptive management (p < 0.001). Deep bite showed a moderate but significant association with borderline treatment need, particularly in the absence of traumatic gingival contact (p = 0.002). Open bite, though the least prevalent, was significantly associated with a definite orthodontic treatment need, especially in cases with functional impairment (p < 0.001).

The findings demonstrate that increased overjet and crowding are the strongest contributors to the need for definitive orthodontic treatment. At the same time, anterior crossbite and open bite, despite lower prevalence, represent clinically significant conditions that warrant early diagnosis and timely intervention.

## Discussion

The present hospital-based study was conducted to evaluate the prevalence and pattern of malocclusion and its association with various orthodontic treatment needs among children in the mixed dentition period of the Kashmiri population in the Department of Paediatric and Preventive Dentistry, Government Dental College and Associated Hospital, Srinagar (which is the only tertiary care dental hospital in Kashmir Valley), using the IOTN. There is a deficiency in literature regarding malocclusion prevalence in the mixed dentition period of the Kashmiri population, so this was the initial step towards determining its prevalence by doing the tertiary hospital-based study. On evaluating the results of the study, it was found that the overall prevalence of malocclusion was 19.9% which is much lower than reported in various Indian and international studies, where prevalence ranges from 23% to 45% Mehta et al. (23 to 43%), Sandhu et al. (36%) and Alex et al. (28-45%) [2-4]. However, the proportion of children requiring definite orthodontic treatment (12.3%) is comparable with findings reported by studies (Thilander et al., Onyeaso et al., and Kapoor et al.) using IOTN in similar age groups. Such variations could be attributed to differences in age groups, environmental factors, ethnic composition, population characteristics, sampling strategies, diagnostic criteria, and study settings [7-9]. Moreover, hospital-based studies are often expected to report higher prevalence due to selection bias, but our study included all consecutively eligible children attending the outpatient department, many of whom presented for routine dental care rather than orthodontic complaints. Malocclusion was more prevalent in the female population, and these results were in accordance with studies done by Amro et al., NP et al., etc and the reason would be attributed to the fact that parents are more concerned about the aesthetics of their female child and want to go for treatment as soon as possible [10,11].

Among children who were diagnosed with malocclusion (n=1848), increased overjet was the most prevalent trait (33.5%), followed by crowding (29.2%), anterior crossbite (16.8%), deep bite (13.4%), and open bite (7.1%). This distribution was found to be consistent with earlier studies conducted among Indian school children and mixed dentition populations (Siddiqui et al., Tausche et al., Avinash et al.), where increased overjet was the most frequently reported occlusal anomaly [12-15]. This higher prevalence of increased overjet observed in our study was clinically significant, as excessive overjet was found to be associated with increased susceptibility to traumatic dental injuries, lip incompetence, and esthetic concerns, which may be one of the reasons for adversely affecting a child’s quality of life [16,17]. Crowding, which is commonly encountered during the transitional dentition phase, reflects discrepancies between tooth size and arch length, a strong indication for orthodontic consultation and treatment [18]. The presence of increased overjet or crowding is an obvious sign of dental malocclusion, and parents, to some extent, may be aware of their child’s malocclusion status.

Assessment of parameters using the IOTN-DHC revealed that a substantial proportion of children with malocclusion (61.6%) exhibited a definite orthodontic treatment need (Grade 4-5). A highly statistically significant association between malocclusion trait and orthodontic treatment need (p < 0.001) was demonstrated, highlighting the importance of severity and etiology-based assessment rather than reliance on prevalence alone. The significant association observed in our study indicated that increased overjet greater than 6mm and mesiodens-related malocclusion were among the strongest predictors for definite orthodontic treatment needs. Increased overjet was classified under higher IOTN grades due to its association with increased risk of incisal trauma, functional impairment, and psychological impact, which was well supported by previous literature by Richmound et al. [19]. Similarly, mesiodens-related malocclusion, confirmed by clinical and radiographic evaluations, was found to result in delayed eruption, displacement, rotation, and spacing of permanent maxillary incisors, thereby necessitating comprehensive orthodontic management in a majority of cases [20,21].

On the other hand, early interceptive orthodontic treatment requirements, as opposed to immediate comprehensive therapy, were observed to be strongly linked with retained primary maxillary incisors that resulted in anterior crossbite of permanent incisors. If left untreated, anterior crossbite during the mixed dentition era is known to result in aberrant incisal wear, functional mandibular shifts, and unfavorable skeletal growth patterns [22,23]. It has been demonstrated that early orthodontic intervention and timely extraction of retained primary teeth can successfully treat such problems, thus minimizing the development of more severe malocclusions and lowering the complexity of subsequent treatments [24,25].

Despite being the least common malocclusion feature in the current study, open bite showed a substantial correlation with the need for orthodontic treatment. This result is consistent with earlier research (Subtelny et al.) that highlights the functional and aesthetic issues related to anterior open bite, such as masticatory inefficiency and speaking problems. Conversely, deep bites were more frequently linked to borderline treatment requirements, especially when there was no traumatic gingival contact [26]. This pattern has also been noted in previous epidemiological studies.

The percentage of children in the current study (12.3% of the overall population) who clearly require orthodontic treatment is consistent with results from an earlier study (Kaur et al.) using the IOTN in comparable age groups [6,13]. The burden of clinically significant malocclusion requiring treatment seems to be fairly constant, despite regional variations in the total prevalence of malocclusion.

These findings underscore the importance of early screening programs during the mixed dentition period, with particular attention to etiological factors such as increased overjet, supernumerary teeth, and retained primary incisors. Early diagnosis and interceptive orthodontic care can significantly reduce treatment duration, cost, and complexity, while improving long-term functional and psychosocial outcomes [23,24].

There is a deficiency in the literature regarding the initiation of early interceptive orthodontics and a lack of focused research on the prevalence and severity of malocclusion during the critical developmental stages of six to 12 years. Although studies have been conducted in different parts of India, there is a notable gap in data from Northern India, especially in diverse areas like Kashmir. Ethnic generic and environmental factors are unique in the Kashmir Valley. To the best of our knowledge, this is the first hospital-based study to estimate the prevalence of malocclusion in the Kashmiri population using the IOTN assessment. This gap emphasizes the need for localized studies to understand malocclusion prevalence and treatment needs of the study area. These findings will also provide baseline data for planning early interceptive orthodontic services in Kashmir, where such services are limited.

Despite its strengths, the present study has certain limitations that should be considered when interpreting the results. The hospital-based design may limit the generalizability of the findings to the broader community, as children reporting to a dental institution may have a higher perceived or actual treatment need compared to the general population. Additionally, the study relied solely on the dental health component of the IOTN, without inclusion of the aesthetic component, which may underestimate the overall orthodontic treatment need influenced by psychosocial and esthetic concerns. Finally, factors such as oral habits, socioeconomic status, and parental awareness, which may influence the development and perception of malocclusion, were not evaluated. Inclusion of these variables in future studies could provide a more comprehensive understanding of orthodontic treatment demand and utilization. Moreover, future population-based multicenter studies for better generalizability to determine malocclusion prevalence in children of the Kashmiri population are recommended.

## Conclusions

This study provided important baseline epidemiological data on the prevalence of malocclusion, which was 19.9%, and orthodontic treatment needs among children in the mixed dentition period of the Kashmiri population. The present hospital-based study demonstrated that increased overjet (33.5%) and crowding (29.2%) were the most prevalent malocclusion traits. Malocclusion was observed in a significant proportion of children, with many exhibiting a definite need for orthodontic treatment. Increased overjet greater than 6 mm and mesiodens-related malocclusion were strong predictors of definite treatment need, while retained primary incisors leading to anterior crossbite were primarily associated with the need for early interceptive intervention. Such data can assist clinicians, particularly pedodontists, including public health authorities, and policymakers in planning targeted preventive and interceptive orthodontic services (habit-breaking appliances, space maintainers, etc), optimizing resource allocation, and developing school- and community-based oral health programs. Future multicenter and population-based studies for better generalizability (for reduction of selection bias), incorporating both the dental health component and aesthetic component of the IOTN, along with assessment of socioeconomic and behavioral factors, are recommended to obtain a more comprehensive understanding of orthodontic treatment needs in the children of Kashmiri population also early etiological screening and timely orthodontic management are essential to reduce treatment complexity and improve long-term outcomes.
